# The Feeling of Pleasure for Overweight Children during Different Types of Physical Activity

**DOI:** 10.3390/children10091526

**Published:** 2023-09-08

**Authors:** Aymen Hawani, Anis ben Chikha, Mohamed Abdelkader Souissi, Omar Trabelsi, Maher Mrayah, Nizar Souissi, Santo Marsigliante, Antonella Muscella

**Affiliations:** 1 The Higher Institute of Sport and Physical Education (Ksar Saïd), University of Manouba, Manouba 2010, Tunisia; benchikhaanis@yahoo.fr (A.b.C.); mrayeh.meher@gmail.com (M.M.); 2 Physical Activity, Sport and Health, Research Unit (UR18JS01), National Observatory of Sport, Tunis 1003, Tunisia; gaddoursouissi@yahoo.com (M.A.S.); trabelsi.omar@issepsf.u-sfax.tn (O.T.); n_souissi@yahoo.fr (N.S.); 3 Research Unit ECOTIDI (UR16ES10), Virtual University, Tunis 1073, Tunisia; 4 The High Institute of Sport and Physical Education of Gafsa, University of Gafsa, Gafsa 2112, Tunisia; 5 High Institute of Sport and Physical Education of Kef, University of Jendouba, El Kef 7100, Tunisia; 6 Department of Biological and Environmental Science and Technologies (DiSTeBA), University of Salento, 73100 Lecce, Italy; santo.marsigliante@unisalento.it

**Keywords:** feeling of pleasure, overweight, physical activities, traditional motor games

## Abstract

The feeling of pleasure during physical education (PE) could increase with physical activity participation and adherence for overweight children. While traditional games are known to have positive benefits on motor skill development, especially for children with poorer motor skills, and on the body mass of children, little is known about overweight children’s feelings of enjoyment when playing these games. To identify a program of physical activity appreciated by overweight children, we tested the effect of different activities, namely soccer (SO), shot put (SP), and traditional motor games (TMGs), on the feelings of pleasure for 28 overweight male children (aged 12.11 ± 0.63 years, BMI 26.89 ± 0.15 kg·m^−2^) participating in a 3-month cross-over study. To measure affective responses to exercises, we applied a validated feeling scale. The data were collected during eight PE lessons, which were organized for each cycle taught. Post hoc pairwise comparisons revealed a significant difference (Z = −3.195, *p* < 0.01) between the mean feeling score reported after the SO cycle (2.48 ± 0.41) and that after the TMGs (3.04 ± 0.32). A similar significant difference (Z = −3.304, *p* < 0.01) was found between the mean feeling scores reported after the SP cycle (2.27 ± 0.32) and the TMGs (3.04 ± 0.32). There was no significant difference between the mean feeling scores reported after the SO (2.48 ± 0.41) and SP cycles (2.27 ± 0.32). In conclusion, the findings of the present study suggest that TMGs may have beneficial effects on the feelings of pleasure for overweight children. Therefore, TMGs might potentially be considered as an alternative to conventional physical activities.

## 1. Introduction

In recent years, there has been a significant decline in physical activity among high school students [1]. While children have plenty of chances for physical activity during the school period, comprehending breaks, sports, physical education classes, and active travel to and from school [2], studies on the international pediatric population reported that more than half of children fail to achieve recommended levels of physical activity and the compliance rates decrease with age [3,4,5]. Commonly, with age, children attain mobile devices such as laptops and smartphones, which increases screen time and reduces physical activity time, regardless of the family lifestyle in which they grow up [6]. This problem is becoming more urgent, mostly after the pandemic, due to the increase in acquired sedentary habits in child and adolescent populations [7]. Indeed, the periodic lockdown and the spread of online courses contributed to an additional contraction in children’s physical activity, and to an increment in physical inactivity, and consequently, in body weight [8].

In fact, low physical activity and childhood obesity are interrelated [9]. Furthermore, some studies have reported that children with low levels of physical activity and that are overweight or obese have compromised psychological well-being [10,11] and cognition [12]. Conversely, the prevalence of physical activity correlates positively with good feelings and satisfaction and negatively with anxiety, depression [13], and cognitive decline [14]. Overweight children with poor physical fitness are likely to maintain this condition of being overweight, with poor physical and mental health, even into adulthood [15]. Indeed, school-based physical activity interventions can reduce anxiousness, increase resilience, and enhance good mental health and well-being in children and teenagers [16].

However, a small percentage of school-age children and teenagers meet the requirements concerning daily PA level [4]. According to estimates, about 80% of school-age children fail to reach the advocated level of physical activity [6], for which the choice of structured physical activity during physical education (PE) classes has been questioned, especially for children with poor physical fitness or who are overweight [17,18]. 

Regarding PA programs for children, the planned activities should motivate and entertain children, as if children become bored, they stop the program [19].

It is important to set up the activities so that they are very enjoyable, especially if aimed at overweight children. In effect, enjoyment during PE lessons appears to have a mediating effect between autonomy support and performance for overweight children [20]. Furthermore, pleasurable sensations during physical education classes are likely to contribute to the continuation of physical activity over time and subsequent health and fitness benefits for overweight children [21,22]. 

Given the recognized importance of motivation and enjoyment for children’s PE, understanding the relationships between these constructs and PA programs is important for intervention efforts aimed at preventing childhood obesity.

During physical activity, mood state is influenced by various factors such as activity modality, effort, competitive performance, and motivation of athletes [19], but no studies were found on children’s feelings of enjoyment during different PE programs.

Young people choose to participate in the activities they love most, such as playing soccer. Soccer is one of the most popular team sports in the world and it is practiced more in school and out of school. Generally, students playing soccer feel greater commitment and perceived great motor competence towards this sport in physical education [23].

However, the sport could be embarrassing for the students by bringing the overweight body to the fore and putting the children at risk of disapproval by their peers [24]. Furthermore, demotivated students may not have felt supported in their ability to be competent within the PE context, due to the continued focus on effective gameplay and not social aspects such as being a quality teammate who played in a proper way [25].

It is known that the programming of traditional motor games (TMGs) during PE lessons, affects the body mass of overweight children [6], can also influence early child development (moral value, language, physic-motor, social emotional, cognitive, and religion) [26] and would be an appreciation of heritage and culture, and therefore an alternative strategy to promote physical activity and improve physical fitness and health [27,28]. It is believed that TMGs constitute an important resource for PE in at least three ways: epistemologically, they allow us to understand the relationship between motor skills and history, society, and culture; pedagogically, they clarify our options in proposing goals and designing study programs; and didactically, they make us understand the resources we have for teaching PE [26]. Despite all these TG advantages, little is known about overweight children’s feelings of pleasure when playing these games.

Therefore, in order to identify a program of physical activity appreciated by overweight children, it would be important to evaluate the mood during different physical activities, practiced by these children; therefore, the main objective of this study is to evaluate the effects of a team sport (soccer), an individual sport (shot put) and traditional motor games (TMGs) played during PE lessons.

In this study, furthermore, we aim to investigate the effect of traditional motor games on the sensation of pleasure that could be an alternative program that contributes to exercise maintenance and health and fitness benefits [29] in overweight children.

## 2. Materials and Methods

### 2.1. Participants

A minimum sample size of 28 was determined from an a priori statistical power analysis using G*Power (Version 3.1, University of Dusseldorf, Dusseldorf, Germany) based on the *t*-test family (means: the difference between two dependent means). The analysis output showed that a sample size of 28 subjects would be sufficient to identify significant differences (effect size = 0.71, power (1 − β) = 0.95 with an actual power of 95.15) in this study.

Twenty-eight male overweight adolescents (mean age 12.11 ± 0.63 years) enrolled in a secondary school in the Tunis area and selected by sampling participated in this study of convenience.

The study was conducted during mandatory physical education lessons, i.e., two PE classes per week. The inclusion criteria were: (1) all participants attended PE classes with the same teacher; (2) had no previous experience in soccer, shot put, or traditional motor games; (3) there was no illness or injury during the study and in the 2 months preceding the study; (4) no prior cognitive or physical disease; and (5) body mass index (BMI) of the attendees was from 25.0 kg·m^−2^ to <30.0 kg·m^−2^. The exclusion criteria were: (1) no regular presence of students in PE lessons; and (2) participant became sick during the experimental period.

All students were asked not to change their usual diet during the study period.

All participants and parents were informed in advance about the study and its objectives and modalities. Participants’ parents gave signed informed consent prior to the start of this study. Attendees were free to abandon the activity without being required to give any type of account and without being chastened in case of abandoning the study.

The study was approved by the Research Ethics Committee of the University (approval n° 04/2022) according to the principles of the latest version of the Declaration of Helsinki.

### 2.2. Study Design

The present investigation was carried out during the 2022–2023 education season (from October 2022 to mid-January 2023). For 12 weeks, the study was conducted under the same experimental conditions (school field; temperature ranged from 19 to 20 °C; between 10:00 and 11:00 a.m.).

This study is almost a cross-over experimental: the data were collected from a single group of overweight children (*n* = 28) who alternately conducted each cycle of activity of soccer, shot put, and traditional motor games (Table 1). Thus, each cycle of activities included 2 lessons per week, for 4 weeks for a total of 8 physical education lessons.

Teachers were instructed to maintain their normal teaching methods and students were instructed to do everything as normal.

### 2.3. Measurements of Anthropometric Parameters

Weight was measured with a scale (SECA; accuracy 0.1 kg). Height was measured in feet using a SECA scale (accuracy 0.1 cm). BMI is a widely used anthropometric measurement even if it does not provide direct information on body composition (Warner DA, Johnson MS, Nagy TR., 2016). BMI (kg/m^2^) is calculated by dividing weight (kg) by the square of height (m^2^). BMI values were used to define obesity indices (overweight, obesity, and severe obesity) These indices were defined according to the International Obesity Task Force (IOTF) [30]. The anthropometric characteristics of participants are presented in Table 2.

During the shot-put cycle (SPC), students learn the related shot put technique by adopting the translational style (O’Brien style). To address this complex motor skill, 2 sessions a week of 50 min each were scheduled.

Also, for soccer cycle (SOC), we scheduled 8 sessions, to enable the learning of the basic concepts of soccer, such as passing, driving, dribbling, and collective play through small-sided games. 

**Figure 1:**
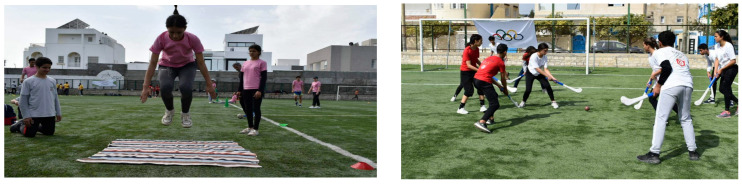

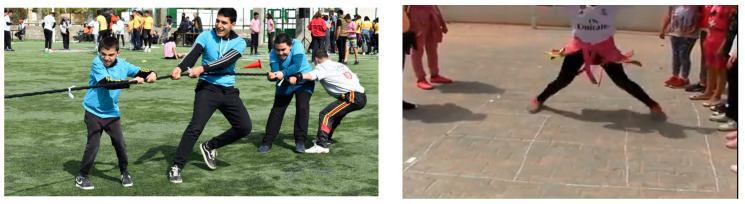
Traditional motor games.

TMGs in Tunisia are very numerous because 24 Tunisian provinces have their own game shaped by the local culture and environment. Among TMGs, we chose 4 for the programming of a traditional motor games cycle (TMGC): Gorguiba (الغرغيبة), Sabâa hajrat (سبعةحجرات), Djeja Aamya (دجاجةعمية), Okfa (العكفة), and traction with the rope (Figure 1).

The feeling scale was performed 5 min post-exercise in every session for 12 weeks. The values were registered immediately after the session and the mean of the measure of affective responses of the two weekly sessions was calculated to obtain a weekly value. In effect, previous recent studies recommended this scale to measure affective responses during exercise [31].

### 2.4. Feeling Measurements

The feeling mood was measured using a feeling scale, that measured the affective response to exercise during the recovery time (realized 5 min post-exercise). The participants answered the question “How do you feel right now?” [32]. 

The feeling scale is an 11-point bipolar scale ranging from +5 to −5, commonly used to measure affective responses during exercise. This scale presents the following verbal anchors: −5 = very bad; −3 = bad; −1 = fairly bad; 0 = neutral; +1 fairly good; +3 = good; and +5 = very good [32]. Before starting the study, instructions of how to properly use the scale were given to the subjects. The values were registered immediately after the session and the mean of the affective responses of the two weekly workouts was calculated to obtain a weekly value. This study protocol has been used in several studies [31,33].

### 2.5. Statistical Analyses

The selection of the appropriate statistical tests was based on how well each collected dataset met the assumption of normality, which was verified using the Shapiro–Wilk test. A dataset was confirmed to be normally distributed at *p* > 0.05. In the case of rejecting the hypothesis of normality, non-parametric alternatives were used.

Mean feeling scores computed after each exposure (eight-lesson in SO, eight-lesson in SP, and eight-lesson in TMG) were analyzed using the non-parametric Friedman’s test. The statistical significance of effects was set at *p* < 0.05. The effect size estimate for Friedman’s test was computed as Kendall’s W, ≥0.1, small effect, ≥0.3, medium effect, and ≥0.5, large effect, by Cohen. Post hoc pairwise comparisons were conducted using the Wilcoxon signed-rank test. As three pairs of datasets were subjected to pairwise comparisons, the threshold for statistical significance (α) was adjusted from 0.05 to 0.017 in adherence to the Bonferroni correction (0.05/3 = 0.017) stipulated in such a case. Effect size estimates for the Wilcoxon pairwise comparisons were calculated as Pearson’s r: <0.3 or −0.3, small effect; <0.5 or −0.5, medium effect, and ≥0.5 or −0.5, large effect.

Data are presented as mean ± standard deviation (SD) in text and mean ± standard error (SE) in figures. All statistical tests were conducted using the statistical software SPSS Statistics (IBM Corp. Released 2019. IBM SPSS Statistics for Windows, Version 26.0. Armonk, NY, USA: IBM Corp).

## 3. Results

**Figure 2:**
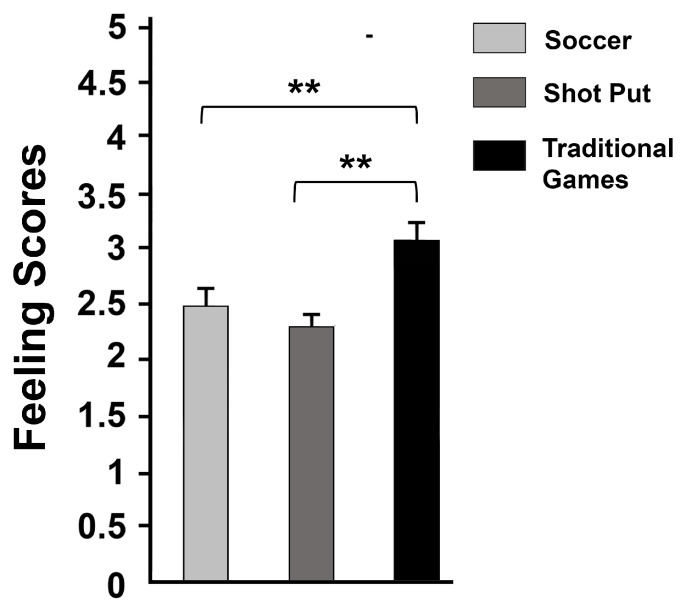
Scores of feelings (mean ± SE) were measured after participating in soccer, shot put, and traditional games cycles. **, significantly different at *p* < 0.01.

The analysis of variance uncovered a significant effect of exposure [X2_F_ = 22.19, *p* < 0.001, W = 0.79] with a large effect. As illustrated in Figure 2, the post hoc pairwise comparisons revealed a significant difference (Z = −3.195, *p* < 0.01) between the mean feeling score reported by pupils after receiving exposure to the SOC (2.48 ± 0.41) and that reported after the TMGC (3.04 ± 0.32) with a large effect size (*r* = −0.85). A similar significant difference (Z = −3.304, *p* < 0.01) was found between the mean feeling scores reported after receiving exposure to the SPC (2.27 ± 0.32) and the TMGC (3.04 ± 0.32) with a large effect size (*r* = −0.88). On the other hand, there was no significant difference between the mean feeling scores reported after receiving exposure to the SOC (2.48 ± 0.41) and the SPC (2.27 ± 0.32).

## 4. Discussion

This study aimed to explore the effect of three activity practices during PE lessons on the feeling mood of overweight children. The results of the present study were as follows: (i) the traditional motor games cycle produced a positive mood (feeling of pleasure) more than the soccer cycle and the shot-put cycle for school adolescents who were overweight; and (ii) the sensation scores for the soccer cycle and shot-put cycle were practically similar.

All over the world, traditional games are an expression of a cultural heritage tradition handed down from ancestors to each generation and preserved according to local wisdom [34]. Traditional motor games, which are simple and very easy make, contribute to non-formal education, creating joyful educational and cultural learning [35,36]. Furthermore, traditional games give character values that can shape individual attitudes to be better in the future [37]; they encourage children to acquire more positive values [38] and to enjoy the pleasure of meeting others [38,39,40,41]. Traditional games enhance values such as a sense of democracy, a sense of responsibility, a sense of friendship, a sense of freedom, a sense of obedience, and mutual help to stimulate interest in children to love companionship and create a pleasant situation for children [42]. Unfortunately, they seemed to disappear and today, they are rarely performed in the community due to various factors such as lack of spaces to play them, gadgets being more attractive than traditional games, etc. [43]. 

The greater sensation of pleasure measured after the participation in TMGs could be explained by the distinctive features of traditional motor games, which have always led people to acquire learning that is as deep as it is enjoyable, being above all, a source of pleasure, fun, and well-being [35,39,44]. These games provoke motor behavior through extremely different exchange systems [45]. It is plausible to assume that TMG proposes possibilities for group decisions that do not favor the establishment of external, unquestioned authorities involving the pupils’ ego, and therefore, that is linked to anxiety and feelings of less enjoyment [46]. Furthermore, these games allow players to enjoy the relational possibilities offered by game situations that are not completely predetermined by other situations [25]. Thus, traditional games are a first-rate pedagogical tool available to physical education teachers to discern the relational tendency of their pupils [47]. 

Since the sensations of pleasure induced by the exercise depend on the participation, adherence, and commitment of the pupils to the activity performed [48,49], these results confirm previous studies showing that enjoyment can also be characterized as a multidimensional construct strongly related to pupils’ enthusiasm and engagement [50]. On the other hand, Louth et al. [51] have revealed that incorporating traditional motor games during physical education lessons can facilitate the involvement of children and motivate students who are less physically active, because they offer children a means to develop motor skills [52] in a climate conducive to learning [46]. This favorable climate and a high level of pleasurable feelings are not always achieved during institutionalized sports [53].

It is known that there is a negative relationship between exercise intensity and feelings of pleasure, and that exercise intensities above the ventilatory threshold (close to the point of respiratory compensation) generate feelings of displeasure [54,55,56]. Because exercise intensities are typically intermittent rather than continuous during TMGs, they are less challenging for participants [57], not affecting their enjoyment of the game. High exercise intensity feelings, low motivation, and frustration affect the activity of overweight children during physical education lessons [58,59,60]; but TMGs produce greater feelings of belonging to the group: it brings back motives for self-determination, creates positive experiences, and minimizes negative emotions. In general, TMGs can play a key role in the emotional facets of physical education [61].

On the other hand, the usual conventional physical activities such as soccer and shot put can be demanding and require greater athletic ability, which may not appeal to less fit pupils. Indeed, conventional physical activities are characterized by being less socially interactive and less autonomous for overweight children during physical education sessions [62]. Other studies support our findings that different types of physical activities may have different associations with feelings [63,64]. However, due to the lack of studies investigating the effect of physical activity, exercise, and sports on mood, a comparison with the current literature is impossible.

We acknowledge the limitations of this study mainly related to the rather small sample size. Further research should address the choice of organizational and communication methods and the variation of teaching styles. In addition, there is a need to move towards studying pupil mood in determining physical activity levels based on several variables, such as geographical area (urban vs. rural), age (primary or secondary school children, etc.), and the genus. However, this may be the starting point for the targeted application of distinct physical activities to foster well-being in childhood and adolescence, and as a useful method to prevent them from being overweight.

It is worth mentioning that childhood obesity is a serious global health problem; the prevalence of childhood obesity was 19.2% between 2017 and 2020 among those aged 2–19 years, thereby affecting approximately 14.4 million children and adolescents (Centers for Disease Control and Prevention [CDC], 2021 [65]). The global COVID-19 pandemic in 2020 increased sedentary lifestyles [66], which placed children at a higher risk of unhealthy weight gain. Obesity is a known risk factor for cardiovascular disease and diabetes, thus responsible for most healthcare expenditures. 

Therefore, teachers should apply this knowledge in their professional contexts, modifying games/sports during PE sessions, to achieve fun and better mood states in overweight children [59]. Further research investigating how TMGs can influence overweight children’s cognitive, linguistic, social-emotional, and physical-motor development, would be desirable. Therefore, teachers should apply this knowledge in their professional contexts, modifying games/sports during PE sessions, to achieve fun and better mood states in overweight children [58]. Further research investigating how TMG can influence overweight children’s cognitive, linguistic, social-emotional, and physical-motor development would be desirable.

The are other limitations to this study that must be taken into account when interpreting the results. First, the study included only male overweight children, which limits the generalization of results. Therefore, future research should consider recruiting children with a larger sample size for both genders. Second, this research is based on the practice of overweight children during PE sessions, and physiological measurements (heart rate, oxygen consumption, fatigue levels, etc.) must be included in future research. Furthermore, it may be useful to associate other variables such as cognitive measures, teaching styles, and geographical location (urban or rural) with this type of research. Considering the inherent limitations of this study, these preliminary results nonetheless show a potential effect of TMGs during physical education lessons on twenty-eight overweight children; such findings need to be validated in future larger, well-designed studies.

## 5. Conclusions

This study builds on prior research and confirms that replacing institutional gaming with TMGs has beneficial effects on post-exercise mood and increases physical activity among overweight children during PE lessons. Also, and not least, TMGs are more enjoyable than SOC and SPC. In effect, TMGs require fewer specific skills, and the purpose of these games is to promote enjoyment rather than competitiveness, especially for overweight children less inclined to participate in sports or competitive games. Therefore, the study results provide PE teachers with a new resource with which to organize their learning content along different lines than those of the traditional play–game–sport continuum, mainly for overweight pupils in PE lessons.

## Figures and Tables

**Table 1:** Representative diagram of the experimental protocol.

	Testing Sessions																							
	SOC								SPC								TMGC							
	Week 1		Week 2		Week 3		Week 4		Week 1		Week 2		Week 3		Week 4		Week 1		Week 2		Week 3		Week 4	
Group (n = 28)	S_1_	S_2_	S_3_	S_4_	S_5_	S_6_	S_7_	S_8_	S_1_	S_2_	S_3_	S_4_	S_5_	S_6_	S_7_	S_8_	S_1_	S_2_	S_3_	S_4_	S_5_	S_6_	S_7_	S_8_

S_n_, PE session number; the background color distinguishes the three activity cycles.

**Table 2:** Anthropometric characteristics of participants (mean ± SD).

Pupils (n = 28) Average ± SD	
Height (m)	152 ± 0.84
Weight (kg)	62.13 ± 0.21
BMI (kg/m^2^)	26.89 ± 0.15

SD, standard deviation; BMI, Body Mass Index.

## Data Availability

Not applicable.
